# In-depth reasons for the high proportion of zero-dose children in underserved populations of Ethiopia: Results from a qualitative study

**DOI:** 10.1016/j.jvacx.2024.100454

**Published:** 2024-02-01

**Authors:** Gashaw Andargie Biks, Fisseha Shiferie, Dawit Abraham Tsegaye, Wondwossen Asefa, Legese Alemayehu, Tamiru Wondie, Gobena Seboka, Adrienne Hayes, Uche RalphOpara, Meseret Zelalem, Kidist Belete, Jen Donofrio, Samson Gebremedhin

**Affiliations:** a Project HOPE, Ethiopia Country Office, Addis Ababa, Ethiopia; b Project HOPE Headquarter, Washington, DC, United States; c Maternal and Child Health, Minister of Health, Addis Ababa, Ethiopia; d USAID Ethiopia Country Office, Addis Ababa, Ethiopia; e Bill and Melinda Gates Foundation, United States; f School of Public Health, Addis Ababa University, Addis Ababa, Ethiopia

**Keywords:** Supply and demand barrier, Qualitative, Gender norms, Key informant interview, Focus group discussion

## Abstract

Increasing attention is being given to reach children who fail to receive routine vaccinations, commonly designated as zero-dose children. A comprehensive understanding of the supply- and demand-side barriers is essential to inform zero-dose strategies in high-burden countries and achieve global immunization goals. This qualitative study aimed to identify the barriers for reaching zero-dose and under-immunized children and what and explore gender affects access to vaccination services for children in Ethiopia. Data was collected between March-June 2022 using key informant interviews and focus group discussions with participants in underserved settings. The high proportion of zero-dose children was correlated with inadequate information being provided by health workers, irregularities in service provision, suboptimal staff motivation, high staff turnover, closure and inaccessibility of health facilities, lack of functional health posts, service provision limited to selected days or hours, and gender norms viewing females as responsible for childcare. Demand-side barriers included religious beliefs, cultural norms, fear of vaccine side effects, and lack of awareness and sustained interventions. Recommendations to increase vaccination coverage include strengthening health systems such as services integration, human resources capacity building, increasing incentives for health staff, integrating vaccination services, bolstering the EPI budget especially from the government side, and supporting reliable outreach and static immunization services. Additionally, immunization policy should be revised to include gender considerations including male engagement strategies to improve uptake of immunization services.

## Introduction

The 2021 WHO/UNICEF global annual estimate reveals a troubling statistic: the majority of children who have not received any vaccinations, also known as zero-dose children, reside in low- and lower-middle-income countries [1], [2]. These nations account for a staggering 87 % of the total 18 million zero-dose children worldwide [3]. Among them, six populous countries namely India, Nigeria, Indonesia, Ethiopia, Philippines, and the Democratic Republic of Congo are responsible for half of all zero-dose children [3], [4]. However, it is important to note that there are regional disparities within these countries, resulting in higher infant mortality rates. Unfortunately, sub-Saharan Africa bears the brunt of this tragic loss, as millions of children succumb to preventable communicable diseases. The solution to this devastating problem lies in immunization, a transformative process that empowers individuals to combat the microbial threats of the world [5].

Immunization is the ultimate shield against infectious diseases, particularly in the realm of childhood health. It not only plays a crucial role in reducing mortality rates worldwide but also has the potential to significantly improve the overall well-being of communities. In fact, a shocking report by WHO between 2001 and 2008 revealed that nine million child deaths globally were attributable to vaccine-preventable diseases, with sub-Saharan Africa bearing a disproportionately high burden. Within this region alone, 4.4 million innocent lives were tragically lost [3].

To address this grave issue, it is imperative to fortify routine immunization services. By doing so, we can effectively curb the spread of vaccine-preventable diseases and protect the lives of millions across the globe [6]. Ethiopia, a nation steadfast in its commitment to safeguarding the sanctity of life, has set an ambitious goal of increasing immunization coverage by 10 % annually. Recognizing the pivotal role of universal immunization in reducing infant and child mortality rates, Ethiopia has made substantial progress in immunizing children against six common and dangerous diseases such as tuberculosis, diphtheria, pertussis, tetanus, polio, and measles [7]. Ethiopia's has also a dedication extends to the introduction of supplementary vaccines that provide comprehensive protection for our children. These additional vaccines shield them from perils such as hepatitis B, Haemophilus influenzae type b (Hib), pneumococcal diseases (PCV), and the notorious rotavirus [8].

Comprehending the intricate interplay of social, economic and political factors surrounding zero dose children is critical in designing effective immunization programs, given their propensity to encounter numerous barriers. Furthermore, it has become evident that factors such as education, occupation, household income, gender, living conditions, habitation and awareness continue to exert substantial influence even when cost-free immunization programs and other health services are available [9], [10]. Existing immunization practices and delivery systems have failed to adequately meet the needs of populations, especially for those residing in underserved setting resulting in lower coverage rates. Despite numerous efforts, the inhabitants living in these areas pose a significant challenge for achieving the national immunization goals. Gender-related barriers contribute to the unequal access to vaccinations among children. Research shows that empowering mothers play a significant role in reducing the number of unvaccinated or zero dose children. Specifically, a survey-based women’s empowerment index indicates that children with empowered mothers are far less likely to be zero-dose [11]. The index measures different aspects of empowerment, including social independence. It revealed that children of mothers with low or medium levels of social independence were more likely to be zero-dose than those with highly independent mothers [12]. Addressing the barriers to immunization associated with women’s empowerment could lead to a substantial decrease of 4.7 million zero-dose children globally. Consistent with the literature on inequalities in access to various health services [13], children from poorer households are more likely to be zero-dose than children from wealthier households [14]. Unfortunately, there appears to have been little progress in reducing this gap over the past decade and the greatest absolute inequalities occur in the poorest countries [15]. While efforts have been made to overcome these inequalities, progress remains limited. The current situation highlights the need for targeted interventions to ensure equal access to immunization for all children regardless of their socioeconomic background.

Employing a multi-level qualitative approach offers a unique opportunity for real time interactions with stakeholders including beneficiaries, healthcare service providers, policy makers, and influencers who form a principal element of any immunization program. This approach enables a comprehensive understanding of their unbiased perspectives, which have the potential to influence immunization program coverage or other desired outcomes. Evidence generated through this approach is critical for identifying the underlying potential drivers, parental concerns regarding immunization of their children, specific needs of the underserved settings, and analyzing the levels of influence on health-related behaviors [14], [15], [16]. Unlike most studies are based solely on quantitative approaches, which have inherent limitations in capturing nuanced perspectives, a multi-level qualitative approach allows for a deeper understanding of the various stakeholders and complex contexts within the community that impact the coverage or other desired outcomes related to the immunization program [10], [17] for example, interventions such as reminders or recall systems, financial incentives, reducing the physical distance to health services and regular monitoring can serve as powerful catalysts for accelerating immunization services [18]. By embracing this approach, we gain valuable insights that inform targetted strategies to promote immunization and address challenges faced by different communities.

One significant gap in the evidence base about zero-dose children lies in understanding the patterns and challenges faced by refugee, migrant, and nomadic populations [19]. These populations are constantly on the move, and their size can be influenced by conflicts, climate shocks, food shortages, natural calamities, and loss of income [20]. Consequently, accessing immunization becomes even more difficult for zero-dose children and their families who already face multiple deprivations related to health and development [21].

To address this gap, our study aims to explore supply and demand-side barriers that hinder efforts to reaching zero-dose children in underserved settings of Ethiopia. By exploring the unique context of these settings, we aim to shed more light on how gender norms, roles and relations affects vaccination service provision and utilization within the same study settings.

## Materials and methods

### Study design

The study employed a qualitative design as part of a national zero-dose evaluation project to explore barriers and enablers contributing to the high proportion of zero-dose children in underserved and special setting population of Ethiopia from March to June 2022.The overall findings of the study has been documented separately. The study followed a stepwise qualitative approach, initially seeking input from: high-level officials at national level, and subsequently gathering perspectives from mid and lower-level decision makers and community members in selected underserved settings. Qualitative data were collected through key informant interviews (KIIs) and focus group discussions (FGDs).

### Study setting

In total, the study included 22 districts from nine regions (Afar, Somali, Oromia, Southern Nations, and Nationality People (SNNP), Southwest, Gambella, Benishangul Gumuz, Sidama, and Harari) and two city administrations (Addis Ababa and Dire Dawa) of underserved and special populations in Ethiopia. The second-round qualitative study explored the situation in different study populations (including hard-to-reach, pastoralist, conflict-affected areas, socially disadvantaged urban, refugees, and internally displaced populations). Multiple perspectives were considered, and data was collected at different levels of the health system and communities in those study settings.

### Study participants

The study participants were selected in three levels. In addition, the study also included study participants from the Ministry of Health (MoH) such as EPI team leaders, Directors of Health Extension Program/Primary Health Care, and health systems special support Directorates and their respective counterparts at regional level were also represented. Furthermore, key partners including multilateral agencies and nongovernmental organizations (NGOs) at national and regional level were included. We also interviewed the EPI focal persons at the Ethiopian Pharmaceutical Supply Services placed at central and regional hubs. In addition, community-level respondents including formal and informal community leaders, Women/Health Development Army (WDA/HDA) members and caregivers, were selected in consultation with local Health Extension Workers (HEWs). The study participants were selected using a purposive sampling criteria, focusing on respondents who were more likely to provide valuable and insightful information (Table1).

**Table 1:** Summary of the study participants for Barrier analysis for zero dose children, in underserved and special setting population of Ethiopia from March to June 2022.

List of study participants
**Federal-level respondents (# 14 KII)** MoH, MCH Directorate Deputy Director MoH, EPI Team Leader MoH, Primary Health Care (PHC) Technical Advisor MoH, Policy and Planning, Monitoring and Evaluation Directorate Director MoH, Health System and Special System Team Leader MoH, Disease Surveillance and Response Directorate Director EPHI, Disease Surveillance and Response Directorate Federal EPSA, Vaccine Supply Manager

**EPI Focal persons at core partner organizations (UNICEF, WHO, CHAI, PATH) Regional-level respondents (# 84 KII)** RHBs, MCH Director RHBs, EPI Team Leader or equivalent RHBs, HEP Director Regional EPSA Hubs, EPI Focal Person Regional EPHI, PHEM Director Local partners (2/region): UNICEF, WHO, CDC, JSI, CHAI, Save the Children, Transform PHCU

**Zonal or sub-city level respondents (# 36 KII)** MCHN Directorate Director EPI Team leader

**Woreda-level respondents (# 66 KII)** MCHN Directorate Director EPI Team Leader HEP Directorate Director Local implementing partners/ NGOs

**Primary Health Care Unit (PHCU) (# 88 KII)** MCH/EPI head (Primary hospital) HEW supervisors (health centres) MCH/EPI head (health centres) HEWs

**Community-level respondents (# 39 KII, 22 FGDs)** Kebele administrators Influential community members Women Development Army/Health Development Army (WDA/HDA) Local women/caregivers

**Refugees and IDPs (# 25 KII, 10 FGDs**) Humanitarian agencies working in conflict areas/IDPs or refugees. Health workers at refugee/IDP camps Community Health Agents (CHAs) at refugee/IDP camps

### Data collection

Data were primarily collected through KIIs and FDGs in two phases. The first phase was completed with the situational analysis phase in March 2022. This phase captured the perspectives of core partners of the national immunization program and top-level officials from MoH and key-informants from all regional states of Ethiopia excluding Tigray. The second phase involved gathering view points of decision makers including zonal and woreda health offices managers, implementing partners, and health workers deployed at all levels of the primary healthcare unit. The second phase also assessed the perspectives of the community through conducting in-depth interviews with formal and informal community leaders and FGDs with caregivers of children in underserved settings. This phase was completed in June 2022. To ensure consistency of the interviews and discussions, the KIIs and FGDs were facilitated using semi-structured guidelines were used and were based on 12–15 open ended and exploratory questions. The facilitators of the interviews held PhD qualifications and were trained and experienced while the note takers had an MPH as qualification. The interviewers were recruited from local universities from the respective regions based on their merits.

### Data analysis

Data from KIIs and FGDs were analyzed using thematic analysis. The audio recordings were transcribed, and translated from five local interview languages to English. All KIIs and FGDs were tape recorded. Coders independently coded each translated transcript, which was then reviewed collaboratively to establish standardized themes and codes for the analysis ensuring inter-coder reliability. For better analyses, the transcripts for KIIs were first analyzed for exploratory reasons to learn more about vaccination hindrances and the general usage of health services. This approach helped to distill important preliminary issues regarding study themes. After this, transcripts from FGDs were analyzed to complement findings from KIIs. This approach helped to look at vaccine hindrances from a broader perspective, giving a more holistic explanation of low vaccination coverage by capturing the voices of women and community leaders who did not participate in the KIIs and may have had a different perspective.

### Data qualitative assurance

To ensure data quality, interviewers underwent a two-day training in Addis Ababa. Data collection continued until information saturation is achieved. Verbatim translation and transcription were conducted, and the resulting data along with field notes were sent to the researchers for feedback and analysis. The data collection tools were also validated by stakeholders including MoH and regional health bureaus (RHBs). Developing themes were identified, and the report was organized in logical order according to the themes and sub-themes of the analysis. Key quotations were provided to support the interpretation and demonstrate how the findings evolved from the actual data.

The validity of the qualitative research was ascertained by ensuring that data collection and analysis approaches were compatible with the five-dimension criteria (credibility, transferability, dependability, confirmability, and reflexivity) set for assuring rigor of qualitative research [22], [23]. To ensure the credibility, we triangulated the data coming from different levels (high, middle, and low managers) and types (government bodies vs partners, health workers vs community representatives) of respondents. Findings were also validated with selected key informants, and a two-staged validation workshop was conducted with the core research team and those who contributed to data generation. This process helped assess the transferability and confirmability of the findings by presenting them alongside contextual data such as the setting and type of population.

### Ethical considerations

Before data collection, we obtained ethical clearance from the Institutional Review Board of the Ethiopian Public Health Institute. Administrative clearances were obtained from various levels of the health system. Consent was obtained from each participant before they could join the study. To prevent the risk of COVID-19 transmission, precautionary measures including use of hand sanitizers, face masks, physical distancing, and ventilation of interview settings were practiced. Furthermore, the data were used solely for this study and not shared with any third party.

## Results

A total of 368 KIIs and 33 FGDs were conducted in the study. To provide a comprehensive analysis, the study employed the EPI component as the main thematic framework and further customized using WHO health system building blocks to summarize the underlying reasons for the significant number of zero-dose children in underserved settings of Ethiopia as follows;

### Service-delivery related barriers

#### Hard-to-reach areas and lack of road infrastructure

One of the major supply-side barriers that hinders provision of vaccination services is physical inaccessibility of health facilities for the target population in the study areas. The issue is particularly severe in the four pastoralist and developing regions and the newly established Southwest Ethiopia People’s region. In addition, inaccessibility of health facilities due to lack of road infrastructure and topographic barriers were also reported in SNNP, Oromia and Amhara regions. *“Health workers cannot reach the pastoralists over all that distance.”* Key informant from Ethiopian Orthodox Church Development and Inter-Church Aid Commission (EOC-DICAC)

*“Pastoral communities remain inaccessible for 3*–*4 months during the dry season”* Key informant from Hammer WoHO, SNNP region

Regions like Sidama and Amhara, remote areas are too challenging for female HEWs. As reported from Amhara region, female HEWs usually avoid traveling to hard-to-reach areas due to fear of sexual violence and because its physically challenging to them and they fear sexual violence. “*HEWs are afraid to go these remote villages because they are women*” Key informant from Debark WoHO, Amhara region “*The HEP has to engage both male and female HEWs. It is not possible to cover hard-to-reach areas through female HEWs alone*” Key informant from Semen Mountain Mobile Medical Services Organization

#### Vaccination service delivery platforms

##### Static vaccination service

Despite MoH‘s direction to provide daily vaccination services through the static approach, the availability of these services still remains limited. For example, a key informant from Amhara RHB stated that *“We have recently identified that there are health centers and health posts which do not give static immunization service.”*

In many settings static service is not being provided on regular basis due to closure of health facilities and unavailability of health workers, shortage of refrigerators, frequent campaign-based activities, and schedule-based EPI service provision.

A key informant from Agew Awi ZHD, Amhara region stated, *“Our target is to provide static vaccination service at least once per week at health centers and health posts level that have refrigerators.”* Another key informant from a health center in Dire Dawa city Administration also mentioned that *“We [health professionals at the health center] provide static vaccination service on the 15th and 16th day of the month; whereas they [HEWs] provide outreach service from 17 to 20 days of the month”.*

The single most important barrier to provide static vaccination service is shortage of refrigerators at health posts. Across most regions (excluding urban settings), many health posts do not have their own refrigerators, and vaccines are stored in the nearby health centers.

*“In those health posts having no refrigerator, it is not possible to deliver the static services on regular basis”* said a key informant from Loko Abaya WoHO, Sidama region*.*

Conversely, in Afar and Somali regions the static approach is more actively used due to the weak Health Extension Program, unavailability and demotivation of HEWs, absence of refrigerators and interruption of vaccine supplies. In these regions, static service provided at health centers level is the only functional approach to deliver vaccination services.

*“In our district there are two health centers. The vaccination coverage is better in areas located near to the health centers. Elsewhere, the coverage is low [because the health posts are not functional]”* share one key informant from Kori WoHO, Afar region, and another key informant from Gamo ZHD, SNNP region added that *“In the zone, we have 57 health centers; among these only 36 health centers provide child vaccination services.”*

In major urban areas including Addis Ababa, overcrowding and long waiting time cause dissatisfaction and discourage caregivers from vaccinating their children. The existing open vial policy that requires certain number of children to be available for providing a BCG or measles vaccines is also a major cause of dissatisfaction among clients. In many settings multidose vaccines are only provided on selected days further impacting client satisfaction.

*“In the outreach site, HEWs tell mothers to wait until the required number of children are available to open a vial. At the end of the day, if adequate number of children are not available, they will be told to come back on some other day” WDA member, Guangua woreda, Amhara region.*

#### Outreach vaccination service provision

In general, the provision of outreach vaccination services are faced with challenges, leading to its frequent cancellation in most settings (including urban settings like Dire Dawa city Administration and Harari region). These challenges include the lack of transportation service to distribute vaccines to health facilities and shortage of human resources to provide immunization services. In addition, there are no established mechanisms to cover the expenses, including fuel and per diems for health workers.

Key informant from Gikawo WoHO, Gambella Region stated *that “At district level there is no budget to implement outreach program. In some districts an NGO was covering the expense. Now the program has phased out.”* Key informant from a partner organization working in SW region *“About 20*–*30 percent of health facilities [in Southwest region] have motorcycles, but they do not have budget for fuel”.*

Outreach service also lacks regularity because of lack of commitment and demotivation of HEWs due to financial constraints and lack of incentive mechanisms. In Sidama and Amhara regions, outreach activities are not being scheduled regularly because of negligence of HEWs. In remote districts of Gambella, outreach sessions are organized on quarterly basis. Unfortunately, even when appointments are scheduled with mothers, HEWs sometimes fail to appear at the outreach sites resulting in dissatisfaction.

*“We sit here [at the outreach site] and wait for the HEWs. They may or may not come”* FGD discussant, Yeki woreda, SW region and WDA member, Guangua woreda, Amhara region also stated that *“In the outreach site, HEWs tell mothers to wait until the required number of children are available before opening a vial. At the end of the day, if adequate number of children are not available, they will be told to come back some other time.”*

Key informant from Afder ZHD, Somali region mentioned *“When we talk about why outreach and mobile programs are not available, the answer is simple: there is no budget and the biggest challenge in any district is the same.”*

*“Our responsibility is to provide service at the health center. For any fieldwork we have to be paid (…) Due to lack of budget, we did not provide outreach service for more than two years”* Key informant from a health center in AACA *“AACA Health Bureau provides vaccination service primarily through static approach. That is why we have not been able to reach the informal settlements and slum areas.”*

#### Mobile vaccination service provision

The provision of mobile vaccination services in pastoralist settings such as Somali and Afar regions and some of the districts in Oromia, SNNP, Southwest and Sidama regions, faces significant challenges. Currently, there is an absence of an effective service delivery modality to reach pastoralist and semi pastoralist communities. Integrated mobile outreach strategy is not being implemented due to shortage of vehicles, fuel, budget, limited number of mobile teams, irregularity in field deployment, and unmanageably large catchment area in Oromia (Borena and Guji) and Gambella (Neur and Agnuak zones). Similarly, limited experienec exists in implementingthe mobile strategy in SNNP, Southwest, and Amhara regions.

Key informant from Loka Abaya District Health Office, Sidama region stated that *“The communities are semi-pastoralist. But we only provide static and outreach service. We do not have any mobile vaccination service.”*

Key informant from a partner organization working in Oromia Region *“Mobile teams have been established to serve hard-to-reach areas. However, as Oromia is wide, we could not be able to secure adequate resources for the strategy. Consequently, we were forced to prioritize extremely inaccessible areas. Even there is no resource to support this too.”*

Another key informant from Somali region added *“Unless there are partners that can support the mobile service, it is not possible to provide service through this approach. We do not have any resource to support it.”*

#### Catchup vaccination

In general, many children in hard-to-reach and pastoralist settings have not received vaccination as per the schedule due to a multitude of supply and demand side barriers. Key informants both from the government and partners sides emphasized that catchup vaccination should receive better attention. On a positive note, UNICEF and other partners are supporting the MoH in the development of the guideline and (Post-Immunization Reaction Investigation) PIRI is also being utilized to “catch up” on missed vaccinations in hard-to-reach areas.

According to a key informant from Harari region, vaccination services have been affected due to engagement of health workers in various campaigns, leading to health posts being closed and missed opportunities for vaccinations. According to key informant from Harari RHB stated that *“There were many missed children due to the campaigns (…). This is because when there is a campaign, HEWs would be entirely engaged and health posts get closed, during community Based Health Insurance CBHI campaign the whole staff was out.”*

#### Engagement of public hospitals in EPI services

In most regions, distinct vaccination catchment areas are assigned to primary hospitals to provide immunization services through organizing static and outreach services. Currently, many hospitals only provided selected antigens (BCG and OPV-0) and their services are limited to specific hours and selected days of the week. Even failure to provide BCG and OPV-0 to babies born in the hospital has been reported because of poor coordination system between EPI and delivery units. In general, the major misconception, hospitals frequently assume that vaccination is the duty of health posts and health centers.

Key informant from South Omo ZHD, SNNP region mentioned that *“Hospitals and health centers consider that vaccination service provision is the sole responsibility of the health posts. Only 10 % of the vaccination service is provided by hospitals and health centers.”* Another key informant from SNNP RHB indicated that *“Some health centers do not provide immunization services because they assume vaccination is the duty of health posts.”*

#### Demand-side barriers

While widespread resistance to childhood routine vaccination has not been reported in most regions, there are certain exceptions such as TT and HPV vaccines targeting adolescent girls and Covid-19 vaccines for adults. These vaccines, particularly the first two have been frequently associated with unfounded concerns about infertility.

In most FGDs, mothers highlighted that they used to harbor doubts the importance of vaccines and held various misconceptions in the past. However, these knowledge gaps have gradually improved. For instance, in Sidama, there was a tradition of using a traditional herb “Hamessa” to protect infants from illness. But nowadays, the practice of vaccinating children has become more accepted and is increasingly becoming a norm.

*“Previously mothers used to resist vaccination claiming that “Jesus is their vaccine.” Now, after understanding that vaccinated children are less likely to suffer from diarrhea and pneumonia, the resistance is declining” Key informant from Chire WoHO, Sidama region.*

The demand of the community to vaccination service provided through statistic service is unsatisfactory as they believe they have missed out on certain benefits that were previously available.

Key informant from Turmi Health Center, SNNP Region explained that *“We used to vaccinate children as their parents receive food aid. Later on, mothers who do not get flour start to reject vaccination.”*

While many caregivers understand that vaccination prevents diseases, there is a lack of in-depth knowledge. Only a few are able to list vaccine-preventable diseases beyond measles and polio. Consequently, Caregivers also lack comprehensive understanding of side effects of vaccines and struggle with knowing how to address them when they occur.

*“[when infants] face side effects like fever after vaccination, mothers avoid vaccination in the next appointment day”* Key informant from Amhara RHB*.*

*“Sometimes when we mobilize the community for vaccination, few mothers hide their children fearing that they may develop fever if vaccinated.”* WDA member, Debark Zuria Woreda, Amhara region

*“My child was sick for two days after taking vaccination. I decided not to vaccinate him again.” FGD discussant, Gardamarta woreda, SNNP region.*

In the Amhara region, one prevalent practice that has been commonly reported is that mothers tend to delay vaccination until the infants gets baptized. This is often due to the fear of potential side effects. These demand side barriers are consistently reported across various settings. In urban areas, educated parents resist booster doses provided through campaigns by justifying that the child has already been vaccinated.

Another demand side barrier arises from the challenge of remembering immunization schedule, particularly among illiterate caregivers who face significant domestic workload, and this poses a significant constraint on the effectiveness of the vaccination program. In Afar, Somali and Borena of Oromia region, the hostile climate restricts movement of caregivers who seek health services for their children, indirectly affecting vaccination rates. In Somali, Afar, and parts of SW regions, even though clan leaders are highly influential in their respective communities they have not been adequately engaged in promoting vaccination. In southwest, Sidama and Amhara regions, elders and grandparents exert negative influence on vaccination.

*“At times grandmothers advise mothers not to vaccinate their children by arguing that the children have already been vaccinated by God” HEW, Chire Woreda, Sidama region.*

### Health workforce

While there has been an increasing number of health professionals, shortages of health workers persist in most regions, excluding Addis Ababa city Administration, Harari, and Dire Dawa city Administration. The regions of Afar, Somali, Southwest, and Gambella, particularly face critical shortages, weak academic backgrounds, and major skills gaps in providing health services, including EPI.

Key informant from Gamo ZHD, SNNP region *“Among the ten kebeles in Gardamarta district, only four have HEWs. In the remaining kebeles we don’t have HEWs.”* Another key informant from Surma WoHO, Southwest region added that *“Out of the 17 HEWs we have, only four are diploma holders. The rest are sixth grade complete HEWs who have major skill gaps.”*

Experienced health workers leaving the system further exacerbates the problem, as the transfer of skills become weak resulting in a decline in the quality of the immunization services. As reported from SNNP region, experts at zonal and district health offices offered training opportunities by their respective offices and leave their position immediately after completing their trainings.

Key informant from Afar RHB *“There is a high staff turnover. A health post which was functioning before two months could not be functioning now. The facility may stay closed until new health workers are hired.”*

In all the regions HEWs are becoming increasingly demotivated, reluctant, and resistant to implement health programs with the expected quality due to workload, lack of incentives, partiality in career development opportunities, and working for several years in the same setting without change. They also do not get close support by health centers and district health offices. Specially, in relation to the vaccination program, HEWs engagements in demanding activities like tracing of defaulters, provision of vaccination service per schedule and identification of pregnant women in the community is declining.

Key informant from Gambella RHB mentioned *“There are areas where the motivation of health professionals is very low. Sometimes vaccines are not given despite the availability of supplies.*”

Another kebele leader, Ewa woreda, Afar region highlighted *“In the past, health workers used to travel by camels to provide vaccination service in remote areas. But now there is no health workers’ motivation to provide the service in areas not accessible by cars.”*

Key informant from Debark Zuria WoHO, Amhara region indicated *“At kebele-level many activities of the local government are implemented through HEWs. These are not only engaged in health-related activities, but also in agriculture, education, and political networking.”*

Another key informant from Oromia RHB *“Many HEWs have worked at the same health post for about 7*–*9 years. Initially they were very motivated. But now most of them have established families and are not committed as before.”*

Health workers’ from Gambella, Somali, and Afar regions demand for unjustified benefit and becoming reluctant to be involved in mobile, outreach and demand-creation activities without receiving payments. As health workers are receiving per diems in PIRI and vaccination program led by NGOs, they expect the same in other activities.

*“Previously, when we go to the community for vaccination, we used to be paid at least 15 birr. Nowadays, we do not even get a penny”* Regional MCH department representative.

Now days, due to weak staff controlling mechanism many HEWs reside in towns and unavailable on duty. Reportedly, HEWs frequently fail to appear at health posts or outreach sites despite appointing mothers for vaccination. In many settings, even health centers, do not open on time to serve their clients.

Key informant from Gambella region stated *“There are many health professionals who receive salary but are not available on duty (….). It is difficult to take administrative measures because the issue can get ‘politicized.’”*

Another key informant from Teltele Woreda, Oromia *“Many HEWs leave their workplace on Friday to towns where they live. They return back on Monday. So, they provide service only from Tuesday to Thursday.”*

In addition, a key informant from Gambella RHB *“One of the major problems with HEWs is that they are not regularly available at health post (…). Caregivers bring their children for vaccination, but no one is available to give the service.”*

In general, biomedical technicians are available at zonal and district levels in all regions. However, scarcity of senior technicians was reported in Sidama, SW, Harari, Afar and Somali regions. In most settings, refrigerators are not being maintained timely due to shortage of skilled biomedical technicians at districts level, and lack of timely response by officials.

*“Once a refrigerator is broken, it is very unlikely that it would be properly maintained. The technicians have a serious skill gap.”* Key informant from Gulele Sub-city Health Department, Addis Ababa City Administration.

### Leadership and program management

Leadership commitment is a critical factor in the implementation of EPI programs. According to key informants from partner organizations and lower level health systems, there are instances where the focus of the health system shifts from one program to another based on the contemporary situation and support from donors. For instance, in Amhara region, the ongoing conflict has resulted in reduced the budget allocated for the health sector and limited availability of vehicles for field programs. Similar challenges have been reported in Afar and some districts of SNNP region. In pastoralist settings of Somali and Oromia region, more attention is given to food aid and outbreak response. In specific areas like Loka Abaya and SNNP, the focus is primarily on malaria control.

*“If there is a budget coming for Polio campaign, we use it for nutritional screening, deworming, and vitamin A supplementation service.”* Key informant from South Omo ZHD, SNNP region

*“There is a fund coming from [….] to support EPI Equity program. This fund is meant to cover the per-diem, fuel and other costs required for vaccination program. However, this money has been used partially for other programs”* Key informant from Gamo ZHD, SNNP region.

#### Partnership and engaging influential community members

Compared to other programs, there is greater interest and support from partners at all levels of the health system towards the EPI. GAVI is the major donor covering four-fifths of the national budget for vaccine procurement. In addition, GAVI has also initiatives related to data quality, human capacity development, and supportive supervision. USAID, Bill & Melinda Gates Foundation, and Rotary International are also important indirect donors. UNICEF is a major/core partner providing technical and financial support to the health system at all levels in all regions. Specially at lower levels of the health system, UNICEF is focuses on activities such as demand promotion, supporting microplanning, M&E and data quality assurance.

*“Vaccination coverage is decreasing; it is not like before. It used to be supported by an NGO, but now the program is discontinued due to security concerns.”*, key informant from Kibish Health Centre, Surma woreda, Southwest region. Another key informant from Gikawo WoHO, Gambella region *“Our district used to be supported by the PIRI program, recently the support has been discontinued abruptly and we do not know why. The RHB said nothing why it has been discontinued.”*

In many KIIs and FGDs study participants mentioned that religious and clan leaders, as well as teachers were highly respected and credible sources of information. However, there is a need for more concerted efforts to engage these community members in the dissemination of vaccination information. Currently, Small-scale practices of community mobilization using clan leaders and elders (Somali region) and religious leaders (Amhara and Sidama regions) have been reported. In North Gondar of Amhara region, vaccination schedules are commonly communicated using religious institutions and outreach days are also aligned with monthly religious holidays.

*“For example, there is a health center guard called Abebe [changed name] who usually walks around the villages to disseminate vaccination campaign message using a megaphone. And the people mock him ‘here comes [Abebe] again.” But the same massage would be taken better if we had used elders and religious leaders”* Key informant from Loko Abaya district, Sidama region. This illustartes the potential for greater involvement of influential community members to enhance the effectiveness of vaccination campaigns.

#### Gender-related barriers

Gender related barriers to children’s vaccination in various regions present a significant challenge. Across these regions, men’s limited engagement and lack of involvement in ensuring their children receive vaccines emerge as a gender specific barrier. This stems from the cultural perception that childcare is primarily the responsibility of women. Typically, taking children to vaccination centers and communicating with frontline health workers is usually considered as the duty of women. Even health workers themselves may perpetuate these gender stereotypes, further discouraging fathers from participating in their children’s health care. In the FGDs, involvement of men in childcare (including vaccination) was frequently described as the culture of the urban community. In Somali region extreme reports like “*men don’t know where children get vaccinated*” and “*vaccination of children is not the concern of men*” have been raised during the FGDs with local women. In Afar and Somali, women are responsible for managing household chores as well as many outdoor activities, making it difficult for them to take their child for vaccination without support from other family members.

*“Usually, men are not aware whether their children have received their vaccines or not. Men have other important obligations that hinder them from taking young children to health facilities.”,* a key informant from Somali RHB.

These challenges are compounded by the patriarchal nature of Ethiopian society, where, women have limited decision-making power and control over resources. Unfortunately, women often need permission and money for transportation from their husbands to take their children to health centers. In most settings (as reported specially from developing regions like Gambella, Afar and Somali) men exert control over their partners (spouses) movements, and this may affect utilization of health services.

Key informant from a partner organization working in Afar region stated that *“Though a woman has good awareness of vaccination, she may not bring her child to vaccination because she may have no money for transportation. The money is in his pocket.”* In addition, Health professional from Somali region *“When we ask mothers to bring their infants for vaccination, they tell us that they have to secure permission from their husbands first. Sometimes they decline to come claiming that their husband was not willing.”*

### Information

#### Planning, monitoring and evaluation

Regarding the vaccination planning process, there was notable discrepancy between top-level decision makers and frontline health workers. While top-level managers claim that woreda-based and micro plans are being implemented, frontline workers report a lack of bottom-up planning at the ground level. Planning is predominantly centralized at the district level, with targets distributed to health facilities based on population conversion factors. The top-down approach has led to concerns that microplans are developed merely for completion rather than being translated into effective practice. Micro plans are developed on semi-annual basis through bottom-up approach with regional variations in the implementation and some regions showing stronger commitment, while others face challenges in engaging community due to weak HEP, lack of commitment and difficulty in remote areas. In Oromia, microplanning is commonly used only for campaigns.

*“Micro plans are usually developed for the sake of completion. HEWs develop micro plans simply because they are required to do so. The plans are not translated into practice.”* Key informant from Agew Awi ZHD, Amhara region.

In addition, HEW, Chire Woreda, Sidama region stated *“We have no role in the planning vaccination activities. The plans come directly from the woreda health office and health center.”*

HEWs and few woreda health officers think that developing micro plans according to the RED/REC approach is bulky, time taking, donor/partner driven and resource intensive. In Gambella and Sidama regions micro plans are not prepared with adequate details intentionally due to negligence and reluctance.

Key informant Gamo ZHD, SNNP region *“We never used the RED/REC strategy for planning. It requires more resources and time.”* In addition, key informant from Hamer WoHO, SNNP region mentioned *“RED/REC strategy has not been implemented. We did not get any training on it.”*

In addition, Key informant from WHO Vaccination Program Officer *“The main essence of RED/REC is microplanning. However, micro plans are not translated into practice due to budget shortage.”*

According to the key informants, the biggest challenge encountered while planning for vaccination is lack of reliable conversion factors for estimating denominators.

Monitoring, evaluation, and learning practices also face challenges due to resource constraint and lack of motivation. According to top level managers, in most regions vaccination coverage is being evaluated by Performance Monitoring Teams. However, top-level managers (especially from developing regions) admitted that review meetings, performance monitoring teams, and supportive supervision are not regularly conducted due to shortage of resources and lack of motivation. The lack of feedback to frontline health workers is identified as a weakness of the M&E approach.

*“We don’t have adequate budget to conduct M&E activities regularly. What we get is not even enough to buy consumables.”* Key informant from Chire WoHO, Sidama region

*“According to the standard, ZHD is expected to supervise the WoHO quarterly, but we didn’t do that. The district should supervise health centers every month, and health center should visit health posts on weekly basis. But in practice this has not been done”* Key informant Sheka ZHD, Southwest region*.*

#### Surveillance of vaccine preventable diseases

The Ethiopian disease surveillance system monitors over twenty epidemic-prone diseases targeted for eradication/ elimination including Acute Flaccid Paralysis/polio, measles, and neonatal tetanus, and other diseases of public health importance. Among vaccine-preventable diseases, measles outbreak frequently occurs in hard-to-reach, conflict-affected and pastoralist communities in Ethiopia. Regional Public Health Emergency Management (PHEM) officers have identified poor vaccination coverage and clusters of unvaccinated children as underlying causes for these outbreaks.

*“Most of the time what we observe in measles outbreak settings is that 70 % or 80 % children are not vaccinated”,* Key informant from PHEM team of Oromia RHB.

*“With regard to monitoring of adverse effects following immunization, PHEM’s mandate is disease notification as it is clearly stipulated in the National Guideline. This is the responsibility of the EFDA not ours.”,* Regional PHEM Officer *and “Previously adequate attention was not given to AEFI. It was not an area of focus, there were no reports and appropriate responses. Currently reporting AEFI is part of our job.”* PHEM Officer from Addis Ababa city Administration.

#### Data quality for decision-making

All groups of respondents agreed that, despite recent improvements, poor data quality remains a major concern. The groups acknowledged that data reported by the vaccination program lacks quality. Common indicators of poor data quality include discrepancy between community-based surveys and DHIS-based reports and/or other administrative vaccination coverage reports.

*“The admin report does not correspond with the actual number of zero-dose children on the ground”* Key informant from Gamo ZHD, SNNP Region and key informant from SNNP RHB also added *“At health post level, what is found in the tally sheet cannot be the same in the logbook.”*

Several causes contribute to poor data quality such as the lack of reliable denominators and value for data, carelessness, skill gaps, data fabrication and lack of accountability. Sometimes higher bodies are also resistant to accept low/unsatisfactory vaccination coverage data, indirectly pressuring health facilities and health workersto revise and resubmit false report.

*“Significant number of districts reported coverage figures beyond the vaccine supplies they received. Unfortunately, I have not seen anyone held accountable for reporting false data”* Key informant from MoH *and “If we find false reporting, we will educate the health worker who committed the mistake. But we are not taking any other measure.”* Key informant from Loka Abeya WoHO, Sidama region

Key informant from Loka Abaya WoHO, Sidama region stated *“Sometimes officials also demand health workers to modify and resubmit reports. Yes, we have to speak the truth.”*

#### Social Behavioral Change Communication (SBCC) and community mobilization

Currently in various regions, there seems to be a lack of consistent and diverse efforts in implementing SBCC to promote EPI. In addition, mobile phones are not commonly used for disseminating health messages and reminders across all regions. But there have been some encouraging reports of translation of SBCC materials to local languages or development of new ones in Gambella, Benishangul Gumuz, and SNNP regions. Moreover, multiple regions have reported the use of local radios as a platform to promote immunization**.***“The community mostly trusts messages from health workers. They do not give weight to messages delivered by us (volunteer community workers)” HDA member, Borena zone, Oromia region.*

*“The problem is, HDAs demand incentives and they do not want to go home empty-handed. With the current expensive living situation, no one serves for free”* Key informant from Gamo ZHD, SNNP region.

It is observed that health workers sometimes fall short in providing comprehensive information about vaccination. This includes crucial details such as purpose of the vaccination, appointment dates for follow up doses, and guidance on managing potential side effects. Notably, the failure to provide information specifically about side effects has been identified as a contributory factor to the discontinuation of vaccinations.

*“One of the major problems is that the health workers do not transfer the five basic key messages to the mothers. This is what we understand from our [field] visits. The key messages are not delivered to mothers for example when she should return…”* Key informant from Afar RHB.

### Immunization financing

The health system allocates limited direct budget for vaccination programs, relying heavily on donor funding to cover operational costs including expenses for training, supervision and transportation. Many key informants expressed concerns about budget shortages impeding their ability to organize trainings, implement micro plans, supply kerosene for refrigerators, fuel and covering per diems for outreach activities.

*“It has been more than 40 years since Ethiopia started the vaccination program. But the program is still donor dependent and does not stand by itself” Regional EPI Focal Person “Otherwise, there is no money allocated for vaccination from the government. (…….) Our role is organizing/ facilitating the partners working in EPI” Regional MCH Director.*

### Medical products/technologies

Lack of functionality of health posts due to shortage of essential equipment such as functional refrigerators were also identified as a major barrier in all regions except Harari, Dire Dawa City Administration (DDCA), and Addis Ababa City Administration (AACA).

*“Most health posts or health centers have non-functional refrigerators. Especially when the SDD refrigerators fail, they remain out of service for long time due to lack of spare parts and qualified technicians”,* key informant from Gambella RHB.

*“We only have two* refrigerators *for the health center and health posts. At least we need four. We do not have adequate storage capacity to receive all the vaccines we need*”, key informant from Chire WoHO, Sidama region *and “During our field visits, we frequently observe that the refrigerators [at health facilities] are fully stocked. The available storage capacity at the lower level is limited”,* key informant from Oromia RHB

Limited access to electricity or frequent power outage are among the challenges of the cold chain system. Power outage occurs due to shortage of fuel, lack of budget to purchase kerosene and absence of automatic power back up system.

*“In our hospital, there are adequate number of refrigerators provided by (….), but we face frequent and prolonged power interruption. Our main problem is electricity (….). It is not possible to use the generator for 24 h.”,* key informant from Klan Hospital, Afar region.

Failure of WoHOs to submit Vaccination Requisition Form (VRF) on time and delay in compiling the VRFs has been reported in all regions. Due to inaccessibility, health facilities in Gambella, BG, Southwest, Afar and Somali regions do not frequently submit their request on time. So, it means, that facility will miss the supply for the next 30 days as PFSA does not provide supplies without request.

*“We gave training on timely reporting of [vaccine] needs. But, due to staff turnover, the outcome is not as what we expected. Still continuous training is needed.”,* key informant from Hawassa EPSA Hub. (see Table 2)

**Table 2:** Codes, subthemes, and themes, zero dose children’s barriers, in underserved, hard to reach and special setting population in Ethiopia, 2022.

Codes	Subthemes	Themes
• Lack of dedicated vehicles and the lack of transportation Shortage of motorbikes and poor maintenance of existing ones, Reliance on camels’ transportation, Unavailability and scarcity of fuel Closure of health facilities and unavailability of health workers, Absence and shortage of refrigerators, interruption of vaccine supplies. Frequent campaign-based activities, and schedule-based epi service provision. Shortage of refrigerators at health posts The weak health extension program, Unavailability and demotivation of hews, Overcrowding and long waiting time lead to dissatisfaction and discourage caregivers from vaccinating their children Existing open vial policy that requires certain number of children to be available for providing a bcg or measles vaccines. multidose vaccines are only provided on selected days further impacting client satisfaction.	Static vaccination service	Service delivery platforms
• lack of transportation service to distribute vaccines Shortage of human resources No established mechanisms to cover the expenses, including fuel and per diems for health workers Lacks regularity because of lack of commitment and demotivation of HEWS due to financial constraints and lack of incentive mechanisms Outreach sites are remote described as inaccessible by the community. HEWS sometimes fail to appear at the outreach sites resulting dissatisfaction by mother/caregivers.	Outreach vaccination services	
• Absence of an effective service delivery modality to reach pastoralist and semi pastoralist communities Integrated mobile outreach strategy is not being implemented Due to shortage of vehicles, Fuel, Budget, Limited number of mobile teams, irregularity in field deployment, Unmanageably large catchment area Limited experienec	Mobile vaccination service provision	
• Recent political instability No nationally agreed guideline for implementing catchup vaccination	Catchup vaccination	
• Geographical barriers hindering access to healthcare facilities. Inadequate infrastructure and transportation systems. Insufficient vaccine supply and stockouts in remote areas.	Accessibility and Availability	
• Services are limited to specific hours and selected days of the week. Misconception hospitals frequently assume that vaccination is the duty of health posts and health centers Failure to provide BCG and OPV-0 to babies born in the hospital. Poor coordination system between EPI and delivery units	Missed opportunities for vaccination. Public Hospitals in EPI Services	
• Shortages of health workers persist in most regions. weak academic backgrounds, and major skills gaps in providing health services. Experienced health workers turnover/leaving the system Staff’s workload, working for several years in the same setting without change declining HEWs engagements in tracing of defaulters scarcity of senior technicians weak HEP	Weak academic backgrounds and Experience	Health Workforce
	Staff storage and workload	
• HEWs are becoming increasingly demotivated, reluctant, and resistant lack of incentives, partiality in career development opportunities, Luck of close support by health centers and district health offices Demand for unjustified benefit and becoming reluctant to be involved in mobile, outreach and demand-creation activities	Motivation and incentives	
• Inadequate health workforce and staff capacity. Fragmentation and inefficiencies within the healthcare system. Lack of supportive policies and financing for immunization programs.	Health System Performance	
• Weak staff controlling mechanism. Lack of Digital signature system for human resource and linking with financial system Low performance monitoring system/Low performance appraisal	Poor control mechanisms	
• Ongoing conflict has resulted in reduced the budget allocated. more attention is given to food aid and outbreak response	Focus of the health system shifts from one program to another.	Leadership and Program Management
• men’s limited engagement lack of involvement in ensuring their children receive vaccines emerge as a gender specific barrier cultural perception that childcare is primarily the responsibility of women Children to vaccination centers and communicating with frontline health workers is usually considered as the duty of women. Involvement of men vaccination frequently described as the culture of the urban community. men don’t know where children get vaccinated Vaccination of children is not the concern of men. limited decision-making power and control over resources women often need permission and money for transportation from their husbands men exert control over their partners movements	Gender-related Barriers	
• Highly respected and credible sources of information Vaccination schedules communicated using religious institutions Outreach days are also aligned with monthly religious holidays.	Engaging influential community members and Informal Institutions	
• Recently the support has been discontinued abruptly. Discontinued due to security concerns	Partnership for Vaccination	
• Inadequate community involvement and participation. Limited trust and confidence in healthcare providers. Insufficient	Community Engagement: Unavailability of means of Transportation	
• Health system allocates limited direct budget. budget shortages impeding their ability Relying heavily on donor funding such as expenses for training, supervision, and transportation	Operational costs	Immunization Financing
• Lack of consistent and diverse efforts in implementing SBCC to promote EPI Scarce resources Audio-visual aids are not effectively utilized Fall short in providing comprehensive information. Failure to provide information about side effects. Mobile phones are not commonly used for disseminating health messages and reminders Use of local radios as a platform to promote immunization Translation of SBCC materials to local languages	Social Behavioral Change Communication (SBCC) and Community Mobilization	Information
• Top-level managers claim that woreda-based and micro plans are being implemented. Frontline workers report a lack of bottom-up planning at the ground level Planning is predominantly centralized at the district level Microplans are developed merely for completion rather than being translated into effective practice Lack of committment and difficulty in remote areas Commonly used only for campaigns. Regional variations and commitment in the implementation challenges in engaging community Developing micro plans based on RED/REC approach is bulky, time taking, donor/partner driven and resource intensive. Micro plan not prepared with Adequate details intentionally due to negligence and reluctance. lack of reliable conversion factors for estimating denominators Face challenges due to resource constraint and lack of motivation supportive supervision are not regularly due to shortage of resources and lack of motivation The lack of feedback to frontline health workers	Planning, Monitoring and Evaluation	
• Vaccination program lacks quality data Discrepancy between community-based surveys and other administrative vaccination coverage reports lack of reliable denominators value for data, carelessness, skill gaps, data fabrication lack of accountability health managers and health workers fail to prioritize record keeping and adequately fill tally and registry books Higher bodies resistant to accept low/unsatisfactory vaccination coverage data, Indirectly pressuring health facilities and health workers to revise and resubmit false report.	Data Quality for Decision-making	
• Frequently associated with unfounded concerns about infertility. Certain exceptions such as TT and HPV vaccines targeting adolescent girls and Covid-19 vaccines for adults traditional herb “Hamessa” to protect infants from illness Low self-initiated demand for childhood vaccination Mothers believed that service provided through statistic service is unsatisfactory. mothers tend to delay vaccination until the infants gets baptized due to the fear of potential side effects Domestic workload Hostile climate restricts movement of caregivers. Clan leaders are highly influential in their respective communities but have not been adequately engaged in promoting vaccination. Elders and grandparents exert negative influence on vaccination. Prevailing beliefs and misconceptions surrounding vaccination. Traditional practices and customs impacting vaccination uptake.	Social factors and misconceptions	Demand-side Barriers Vaccine Rejection and Resistance
• Limited awareness and education regarding the importance of immunization In urban areas, educated parents resist booster doses provided through campaigns by justifying that the child has already been vaccinated Administration of multiple vaccination at once, and a fear of injections Caregivers also lack comprehensive understanding of side effects of vaccines and struggle with knowing how to address them when they occur. Health workers often fail to provide adequate information about potential side effects of vaccines. Mother caregivers believe they have missed out on certain benefits that were previously available. lack of in-depth knowledge Only a few are able to list vaccine-preventable diseases beyond measles and polio	Knowledge gap	
• Shortage of essential equipment and vaccines Functional refrigerators Identified as a major barrier in all regions except Harari, Dire Dawa City Administration inadequate supply of vaccine due to national stockout or procurement delay shortage of vehicles to distribute vaccines timely to all districts unmanageably large catchment areas for some hubs long distance between health centers and health posts shortage of budget to transport the vaccine	Supply interruption	Medical products/technologies
• Districts and health facilities in over- or under-forecasting. Hospitals with no predefined catchment population Occasionally health facilities fail to optimally estimate their vaccine needs due to erroneous conversion factors	Difficulty to correctly estimate vaccine need	
• Failure of WoHOs to submit vaccination requisition form (VRF) on time Delay in compiling the VRFs are also blockages to the vaccine logistic system. Health facilities do not submit VRF timely on monthly basis. Incomplete reporting	Failure to request for vaccines on time	

## Discussion

This study explored reasons for the high proportion of zero-dose children in underserved settings of Ethiopia, Through qualitative KIIs and FGDs, the study identified key factors contributing to the high proportion of zero-dose children including poor counselling skills of healthcare workers, low staff motivation, high staff turnover, poor staff control mechanisms, unavailability of HEWs on duty, poor accessibility of health facilities, inadequate vaccine logistic management system, lack of services integration, restricted vaccine open vial policy, inconsistent immunization service times, lack of information during vaccination days, prolonged waiting time at the immunization sites, lack of micro planning and monitoring, small-scale resistance and lack of male engagement and women empowerment.

These findings are consistent with previous studies conducted in middle- and low -income countries, which have also identified supply-side barriers such as unavailability of vaccinators, long waiting times, limited skilled human resources, poor logistics management systems, and lack of transportation for vaccines and consumables [18], [24], [25], [26]. This study also highlighted issues with the quality of EPI data reporting, as discrepancies between administrative coverage, vaccinator-reported coverage and survey coverage exist. Demand-side barriers were also identified as major contributors to the low immunization coverage and zero dose. These barriers included lack of knowledge, misconceptions, financial deprivation, lack of women empowerment, lack of partner support, and distrust of the health system. Other reasons for the low coverage included long waiting times, forgetfulness among caregivers/parents, inconvenient vaccination times and caregiver workload. In this study, EPI staff frequently reported to be unavailable and high staff absenteeism were major contributors towards low immunization coverage. Similarly, other studies stated that performance of the EPI staff, such as vaccinators are instrumental for improving vaccination coverage and decreasing zero-dose children [27]. This can have a detrimental impact on immunization coverage and to have high number of zero-dose children in the study settings in Ethiopia. This study also linked the poor performance of frontline workers to the unavailability of proper monitoring and weak control mechanisms for them. This is a major weakness of public health facilities in developing countries, where workers are not held accountable for underperforming [28], [29]. The outreach vaccination services that are crucial in targeting unvaccinated children in hard-to-reach and remote areas were found to be inadequate.

On the other hand, the quality of EPI data is questionable, with key issues in reporting. Discrepancies between administrative coverage, vaccinator-reported coverage and survey coverage is a persistent problem. In Ethiopia, where the birth registry system is not available, the issue of an underreported denominator presents a huge problem in estimating true coverage. These findings are consistent with other study finding done in other developing countries [30], [31].

Demand-side barriers to routine immunization services could be major contributors to the failure of a program despite its interventions [32]. Demand side barriers include lack of knowledge, misconceptions, financial deprivation/lack of women empowerment, lack of partners’ support, and distrust of the medical systems. Other reasons include long waiting time, parent’s forgetfulness, inconvenient time, and parents’/caregiver’s workload [31].

The other important small-scale resistances were identified in our study such as influence of religion and culture on the perception and decision-making behavior of mothers or caretakers. This study showed that similar trends existed in the past; however, with increasing knowledge and awareness of the community regarding benefits of vaccination, there has been substantial change in the perception of people, and uptake of vaccination by the religion and influential community leaders. In general, the positive attitude was supported by family, community, and religious leader. In practice, the commitment of the parents to vaccinate their children was high, only hampered by lack of time or adequate information. A published article exploring vaccine hesitancy stated that various attitudes seem to result into specific categories; for instance, vaccine refusal attitude could be as a result of having little or no knowledge about vaccine, lack of trust on the vaccine or it could as well link to financial limitations [33]. In addition, maternal trust on vaccinator was shown to be a factor that influenced the uptake of vaccination by previous studies [34], [35]. Since, women were more involved in taking the children for vaccination, and communities follow certain gender rules; the gender of the vaccinator and hence ability of the mother or grandmother to trust the vaccinator was also important.

Interestingly, gender based difference was not observed in this study in reference to vaccination service utilization which might contribute to high proportion of zero-dose children. There was no difference in immunization coverage by gender of the child. This finding was similar to other studies [11], [36] on immunization. At the household level, women’s lack of autonomy is a major immunization barrier in our study and consistent with a study done in Pakistan, where men and religious authorities heavily influence decision-making [37], [38]. Additionally, the woman’s restricted mobility prevents her from going to unfamiliar areas or where cultural barriers exist [37]. In addition, our study identified attitudes towards decision making regarding the benefits of vaccines is critical to efforts to respond to barriers to vaccine uptake. Several women are still unable to make decisions regarding the health of their children, as decisions are made by their male partners/spouses. This highlights the need to empower women as well as invest in health interventions that focus on couples and not individual roles. For instance, various studies have demonstrated that women who discuss health issues with their spouses and have their partner’s approval on, albeit few, are more likely to seek and utilise health services in a timely manner [39]. In addition, the attitudes of service provider were mentioned as deterrents to vaccinations services use. This finding has been reported in various settings under different health programs [40].

Male partners engagement and women empowerment against immunization is often noted as major problem in this study. A study conducted in Cambodia suggested that women’s decision-making power and autonomy were relevant to maternal and child health outcomes [41]. It is important to carefully consider the social contexts during program design and implementation for child immunization. We need to effectively address socio-cultural contexts by involving the entire community, and not only target mothers and female caregivers but also active engagement of males in the whole process of immunization services. The study also raised the pressing need for women to be empowered to overcome their financial challenges in taking their children to vaccination centers. The data identified low financial allocation from government side and suggested increase government financial gross domestic product allocation to their health sector, consistent with the recommendation in the Abuja declaration [42]. Increased financial resources would enable country to equip and upgrade existing health facilities and to increase their numbers. Targeted resources may motivate and enable staff deployed in remote areas for effective outreach activities to maximize coverage of immunization [31]. In addition, in this study inadequate incentives for vaccinators to facilitate outreach work was a major issue mentioned in this study. This finding was supported by other studies and stated that workers who pay out-of-pocket for outreach work expenses are not reimbursed and has critical impact in the immunization services [43], [44].

The study acknowledged that immunization is a shared responsibility involving community, healthcare service providers, policy makers, and parents who are active participants in the process. Effective communication at different levels and consideration of factors especially at the receiver end is essential to strengthen routine immunization uptake. Thus, there is a need to improve the overall clinic environment and conduct regular training sessions for healthcare workers not only from a technical aspect but also in terms of enhancing their ability to communicate and create confidence in the beneficiaries.

This public private partnership (PPP) must be synergised in times of emergency and expertise to provide timely and sufficient vaccination coverage interventions and PPP in improving vaccination coverage in the underserved communities. It is anticipated that the interventions will contribute to the body of knowledge on models of PPPs for addressing zero-dose immunization service delivery for under-served populations in Ethiopia.

While the study’s strengths included the use of KIIs and FGDs to understand the factors affecting vaccination utilization, it was limited to residents of underserved settings in Ethiopia and health officials and providers within the health system. THowever, the external validity of the results is enhanced by the fact that many residents of remote under-served settings in the country share similar challenges in accessing and utilising vaccination services. Triangulation of responses from different sources including FGDs and KIIs, aimed to provide a more representative picture of the situation.

## Conclusions

Our comprehensive analysis reveals that childhood immunization is not influenced by a single factor alone, but by a combination of multiple factors. To effectively address these issues, intervention efforts should target these multiple factors simultaneously. Importantly, strengthening health systems should be a priority, encompassing improvements in enhancing health worker capacity and demotivation of HEWs should be addressed through providing meaningful financial and non-financial incentives. Furthermore, integration of vaccination services such as maternal child health services food aid program and revised budget allocation for EPI program are crucial steps to consider. By consistently providing reliable outreach and static immunisation services, we can address accessibility issues, inconvenient immunization times, and prolonged waiting time that often discourage parents from bringing their children for vaccination. Expanding the reach of the PIRI program to remote districts should be a priority, involving decentralization and additional resource allocation. Immunization policy should be revised to include deploying male HEWs to reach remote settings, allowing for better coverage and engagement with communities. PPPs Furthermore, in order to strengthen immunization efforts, PPP should be actively encouraged and supported. Collaborating with private entities can help leverage expertise and resources, ultimately improving immunization coverage and service delivery.

## Funding

This research was not supported by any specific grant from funding agencies in the public, commercial, or not-for-profit sectors.

## CRediT authorship contribution statement

**Gashaw Andargie Biks:** . **Fisseha Shiferie:** . **Dawit Abraham Tsegaye:** Conceptualization, Methodology, Project administration, Resources, Writing – review & editing. **Wondwossen Asefa:** Conceptualization, Funding acquisition, Methodology, Project administration, Validation, Writing – review & editing. **Legese Alemayehu:** Conceptualization, Funding acquisition, Project administration, Writing – review & editing. **Tamiru Wondie:** Conceptualization, Funding acquisition, Project administration, Validation, Writing – review & editing. **Gobena Seboka:** Conceptualization, Project administration, Resources, Validation, Writing – review & editing. **Adrienne Hayes:** Conceptualization, Project administration, Resources, Validation, Visualization, Writing – review & editing. **Uche RalphOpara:** . **Meseret Zelalem:** Conceptualization, Funding acquisition, Investigation, Methodology, Project administration, Resources, Validation, Writing – review & editing. **Kidist Belete:** Conceptualization, Funding acquisition, Investigation, Methodology, Resources, Writing – review & editing. **Jen Donofrio:** Conceptualization, Formal analysis, Funding acquisition, Investigation, Methodology, Project administration, Resources, Supervision, Validation, Writing – review & editing. **Samson Gebremedhin:** .

## Declaration of competing interest

The authors declare that they have no known competing financial interests or personal relationships that could have appeared to influence the work reported in this paper.

## Data Availability

Data will be made available on request.
